# Can Elderly Patients With Pancreatic Cancer Gain Survival Advantages Through More Radical Surgeries? A SEER-Based Analysis

**DOI:** 10.3389/fonc.2020.598048

**Published:** 2020-10-29

**Authors:** Danna Xie, Baolin Qian, Jing Yang, Xinya Peng, Yinghua Li, Teng Hu, Simin Lu, Xiaojing Chen, Yunwei Han

**Affiliations:** ^1^ Department of Oncology, The Affiliated Hospital of Southwest Medical University, Luzhou, China; ^2^ Department of Hepatobiliary Surgery, Affiliated Hospital of Southwest Medical University, Luzhou, China; ^3^ Department of Clinical Medicine, Southwest Medical University, Luzhou, China

**Keywords:** Surveillance, Epidemiology, and End Results program, pancreatic cancer, surgery, prognosis, propensity score matching

## Abstract

**Background and Aims:**

In recent years, the best treatment method for pancreatic cancer in elderly patients has remained controversial. Surgery is the main treatment modality for pancreatic cancer. This study aimed to determine whether elderly patients with pancreatic cancer can gain survival advantages through more active and radical surgical treatment and evaluate the best treatment method and potential prognostic factors.

**Methods:**

From the Surveillance, Epidemiology, and End Results program (SEER) database, 10,557 elderly patients (aged ≥65 years) with pancreatic cancer were included as Cohort 1, and Propensity Score Matching (PSM) evaluation was performed to generate Cohort 2 (424 pairs). Overall Survival (OS) and Cause-Specific Survival (CSS) were determined using Kaplan–Meier survival curves, and differences were assessed using the Log-rank test. Multivariate logistic regression analysis and the forest plot of hazard ratio (HR) was made to assess the association between potential prognostic factors, including surgery and different surgical methods, and survival in elderly patients.

**Results:**

We identified 10,557 eligible patients with pancreatic cancer, who formed Cohort 1. The total OS and CSS in the surgery group were significantly higher than those in the non-surgery group (*P* < 0.001). Age, stage (AJCC 8th), grade, lymph node metastasis, radiation, chemotherapy, and surgical methods were independent factors affecting the prognosis of elderly patients. In Cohort 2, Total pancreatectomy (Total PT) had the lowest risk ratio (HR = 0.31, *P* < 0.001) and longest median CSS (18.000 months), while Extension Total pancreatectomy (Ex-Total PT, HR = 0.34, *P* < 0.001) showed the lower median CSS (17.000 months) and median OS (14.000 months). Partial pancreatectomy (Partial PT, HR = 0.46, *P* < 0.001) showed the lowest median CSS (13.000 months) and median OS (12.000 months), although they were still higher than the median CSS (6.000 months) and median OS (5.000 months) in the non-surgery group.

**Conclusions:**

Based on the SEER database, surgical treatment is an independent prognostic factor in elderly patients with pancreatic cancer. Compared with other surgical methods, Total PT can offer elderly patients the best survival advantages. However, Ex-Total PT, a more radical method, does not seem to be the best treatment option for the survival and benefit of elderly patients.

## Introduction

Pancreatic cancer is a highly fatal condition (1). The incidence of pancreatic cancer has been increasing year by year, and it is estimated that this pathology will become the second leading cause of cancer-related deaths by 2030 (2, 3). According to the tissue origin, it can be divided into epithelial and non-epithelial origin, and more than 90% of them are pancreatic ductal adenocarcinoma (PDAC) (4). Existing studies report that risk factors for pancreatic cancer include family history of pancreatic cancer, hereditary pancreatic cancer syndrome, pancreatic complications, lifestyles (smoking, excessive alcohol intake), occupational factors, and so on (5). Pancreatic cancer has “three highs and three lows,” that include high morbidity, high recurrence and metastasis, high mortality, low early diagnosis rate, low effective treatment efficiency, and low 5-year survival rate (6). In addition, it is characterized by insidious onset and atypical early symptoms including abdominal discomfort, fullness, loss of appetite, and weight loss (7). The poor prognosis of pancreatic cancer is still a major problem that plagues humans.

Elderly patients are more predisposed to suffer from pancreatic cancer, and its incidence increases with age (8). Most patients are diagnosed when they are above 50 years old, and the most frequent age of onset is between 60 and 80 years (9). Pancreatic cancer in elderly patients is characterized by rapid progress, late detection, and high degree of malignancy (8). Elderly patients with pancreatic cancer usually have a worse prognostic survival than their younger counterparts due to poor health status or comorbidities (10). Even in elderly patients who are in good physical condition and have few complications, age or cancer-induced cachexia might prevent them from receiving more active treatments (11).

The prognosis of pancreatic cancer is extremely poor, with an average 5-year survival rate of less than 7% (12). It can have distant metastasis in the early stage, and is likely to invade local nerves and blood vessels. Besides, it is easy to develop remarkable resistance to conventional treatment methods such as chemotherapy, radiotherapy, and molecular targeted therapy (12). Surgical resection is the only treatment modality that may offer a potential cure for pancreatic cancer and adjuvant chemotherapy has been shown to improve survival rates (13). The median survival time after surgery is approximately 20.100–28.000 months, but only 10 to 20% of patients have the opportunity to undergo surgery (10). It is of vital to evaluate the tumor *in situ* and the local blood vessels involved, which is the key to determining whether or not it is suitable for surgical resection (14). Previous research found that the prognostic for surgery mainly include age, lymph node metastasis, vessel and nerve invasion, tumor size, and CA19-9 level (15). Nowadays, surgery plays an indispensable role in the treatment of pancreatic cancer, but it is unclear whether elderly patients can gain survival advantages from more active and radical surgical treatment. Therefore, it is challenging to find better treatment methods for elderly patients with pancreatic cancer.

SEER is a long-established and publicly-available resource that allows for population-based surveillance and analysis of all cancers in the United States (16), and the statistical data is of large scale and good quality, which reveals the risk model and trend of tumor (17). In this study, we based ourselves on the SEER database to investigate whether elderly patients with pancreatic cancer can gain survival advantages through more radical surgical treatment and to determine the best treatment option and research on potential prognostic factors.

## Patients and Methods

### Data Source

**Figure 1:**
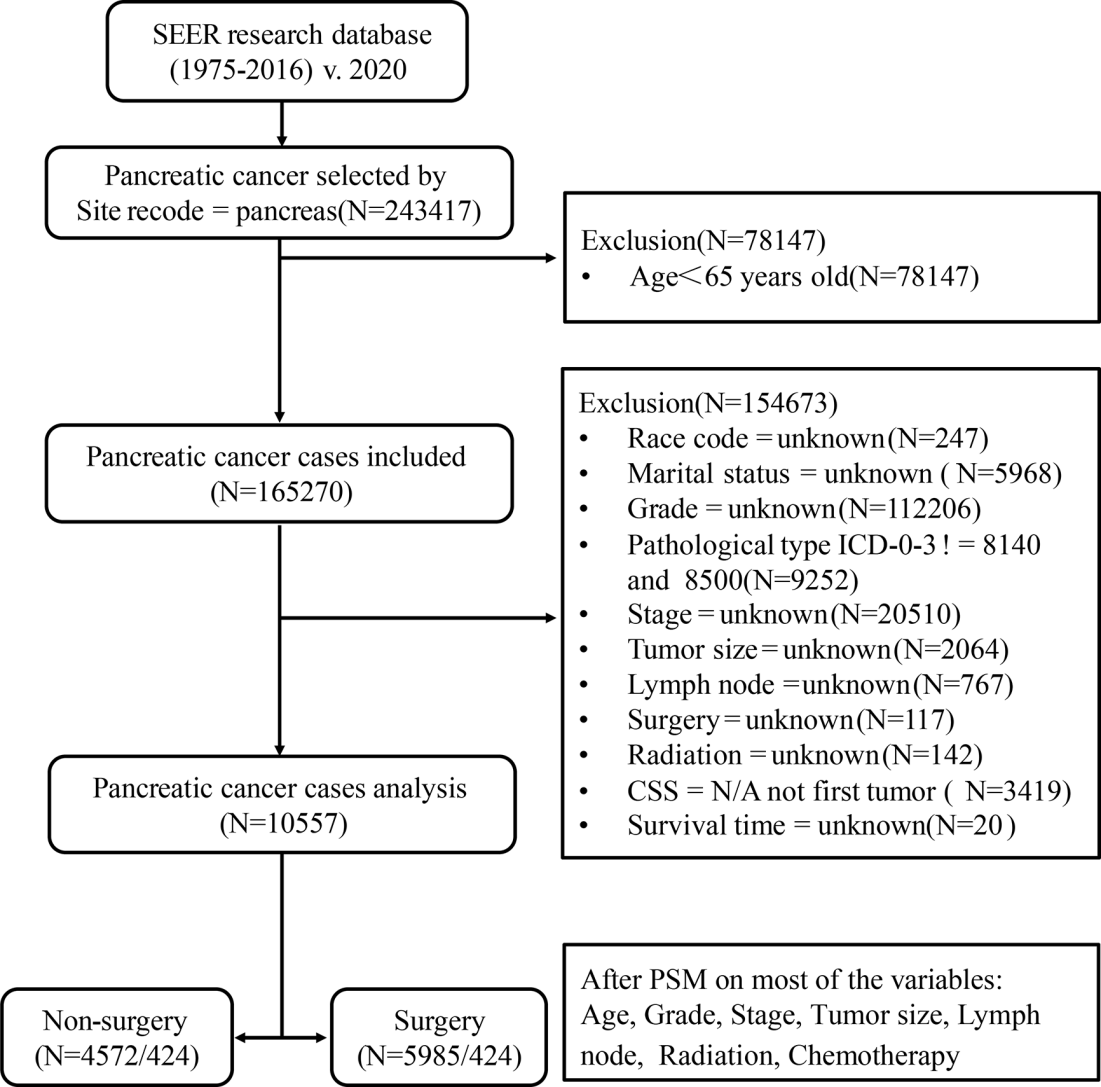
Flow chart of data selection in this study. 8,140, pancreatic acinar cell carcinoma (PACC); 8,500, pancreatic ductal adenocarcinoma (PDAC).

Our study is based on the SEER database (SEER*Stat 8.3.6.1) maintained by the National Cancer Institute, a public, free, and annually updated clinical record platform with demographic and oncology information of cancer patients from 18 registries in the United States (16). We obtained the clinical data of those patients aged ≥65 years, diagnosed with pancreatic cancer (International Classification of Oncology, third edition, ICD-O-3) between 1975 and 2016. The outline of our research design is shown in the flow chart in **Figure 1**.

### Study Cohort

Patients diagnosed with pancreatic cancer between 1975 and 2016, aged less than 65 years old; missing information such as race, marital status, clinical stage, clinical grade, tumor size, lymph node metastasis, radiotherapy, chemotherapy, surgical treatment, *etc*.; those whose follow-up time, overall life status and cause of death are unknown; or those whose pancreatic cancer was neither the only nor the first tumor; or whose tissue type does not belong to pancreatic acinar cell carcinoma (PACC) and PDAC were excluded. Finally, a total of 10,557 patients were included in Cohort 1. PSM is an effective method of adjusting for confounding factors in a retrospective study and improving comparability between groups (18). Based on Cohort 1, we used the PSM function in the SPSS 25.0 software. Patients were then matched in a 1:1 ratio and classified into the surgery group and the non-surgery group. The group variable was selected as surgery. Variables used for matching were those with *P* < 0.001 in multivariate Cox analysis. These included age, grade, stage, size, lymph node, radiation, and chemotherapy. The balances of matched covariates were evaluated with standardized differences. Standardized mean differences before and after matching are presented in **Supplementary Figure 1**. To control for the non-random assignment of patients, we set the Match Tolerance of PSM to 0.000005, which made matched covariates more balanced. After matching analysis, Cohort 2 was formed.

### Clinical Data Preprocessing

According to surgical treatment, the patients were divided into two groups: the non-surgery group (N = 4,572/424) and the surgery group (N = 5,985/424). Then, we regrouped according to the scope of surgical resection, including Partial pancreatectomy (Partial PT), Extension Partial pancreatectomy (Ex-Partial PT), Total pancreatectomy (Total PT), and Extension Total pancreatectomy (Ex-Total PT). Extension pancreatectomy means gastrectomy or/and duodenectomy. The patients included in this study were re-staged according to AJCC 8th. The main endpoints of this study are CSS and OS.

### Statistical Analysis

All statistical analyses were performed with SPSS version 25.0 and R version 4.0.2. Univariate and multivariate Cox proportional hazard models were used to determine the prognostic factors affecting CSS and OS. OS and CSS rates were determined using the Kaplan–Meier survival curve and differences were assessed using the Log-rank test. The forest plot of HR was used to evaluate the association between potential prognostic factors and CSS. All statistical tests were evaluated by using the significance standard *P* < 0.05 (two-sided). HRs were presented with 95% confidence intervals (CI).

## Results

### Clinical Features

In our study, a total of 10,557 pancreatic cancer patients (≥65 years old) were identified from the SEER database. These patients constituted Cohort 1. In Cohort 1, 5,985 patients underwent surgery and 4,572 did not. The mean age of the patients in the surgery group was significantly lower than that in the non-surgery group (73.25 ± 5.80 *vs*.75.30 ± 7.04 years; *P* < 0.001). The baseline characteristics are presented in **Table 1**. After PSM, the distribution of almost all variables of the 848 patients (424 pairs) in Cohort 2 became more balanced; the baseline characteristics are presented in **Supplementary Table 1**.

**Table 1:** Baseline characteristics.

Term	No. of Patients (%)		*P*-value
	Non-surgery (n = 4,572)	Surgery (n = 5,985)	
**Age (years)**			<0.001
Mean (SD)	75.30 (7.0)	73.25 (5.8)	
**Age group**			<0.001
65–69 years	1,191 (26.0)	1,917 (32.0)	
70–74 years	1,076 (23.5)	1,698 (28.4)	
75–79 years	997 (21.8)	1,391 (23.2)	
80–84 years	760 (16.6)	766 (12.8)	
>=85 years	548 (12.0)	213 (3.6)	
**Gender**			0.230
Female	2,462 (53.8)	3,152 (52.7)	
**Race**			<0.001
White	3,645 (79.7)	5,043 (84.3)	
Black	525 (11.5)	442 (7.4)	
Other	402 (8.8)	500 (8.4)	
**Marital status**			0.074
Married	4,143 (90.6)	5,484 (91.6)	
**Location**			<0.001
Pancreatic head	2,489 (54.4)	4,463 (74.6)	
Pancreatic tail	1,329 (29.1)	988 (16.5)	
Other	754 (16.5)	534 (8.9)	
**Grade**			<0.001
Well	565 (12.4)	600 (10.0)	
Moderately	1,800 (39.4)	3,104 (51.9)	
Poorly	2,094 (45.8)	2,230 (37.3)	
Undifferentiated	111 (2.4)	51 (0.9)	
**Pathological type**			<0.001
PDAC	4,122 (90.2)	3,277 (54.8)	
PACC	450 (9.8)	2,708 (45.2)	
**Stage (AJCC 8th)**			<0.001
I	450 (9.8)	579 (9.7)	
II	744 (16.3)	3,570 (59.6)	
III	1,029 (22.5)	1,557 (26.0)	
IV	2,349 (51.4)	279 (4.7)	
**Size**			<0.001
Mean (SD)	43.35 (27.4)	35.02 (18.6)	
**Lymph node**			<0.001
Positive	1,405 (30.7)	3,931 (65.7)	
**Metastasis**			<0.001
Yes	2,363 (51.7)	292 (4.9)	
**Radiation**			<0.001
Received	801 (17.5)	1,930 (32.2)	
**Chemotherapy**			<0.001
Received	2,436 (53.3)	3691 (61.7)	

PDAC, pancreatic ductal adenocarcinoma; PACC, pancreatic acinar cell carcinoma; SD, standard deviation.

### Univariate and Multivariate CSS Cox Regression Analysis

As shown in **Table 2**, the results of univariate and multivariate CSS Cox analyses in Cohort 1 demonstrated that location, pathological type, and metastasis were significantly associated with CSS in elderly patients with pancreatic cancer (*P* < 0.001). In addition, age group (HR = 0.931, *P* = 0.049), stage (AJCC 8th) (HR = 1.239, *P* < 0.001), grade (HR = 1.301, *P* < 0.001), lymph node metastasis (HR = 1.246, *P* < 0.001), radiotherapy (HR = 0.865, *P* < 0.001), chemotherapy (HR = 0.494, *P* < 0.001), and surgical treatment (HR = 0.347, *P* < 0.001) were independent factors affecting the CSS of elderly patients with pancreatic cancer.

**Table 2:** Univariate and multivariate analysis of CSS Cox model before PSM.

Term	Univariate Cox analysis				Multivariate Cox analysis		
	HR	(95% CI)	*P*-value		HR	(95% CI)	*P*-value
**Age (years)**	1.028	1.025–1.032	<0.001		1.028	1.014–1.043	<0.001
**Age group**	1.141	1.122–1.161	<0.001		0.931	0.867–1.000	0.049
**Gender**	0.994	0.953–1.037	0.776		0.956	0.916–0.997	0.036
**Race**	1.027	0.992–1.063	0.132		1.023	0.998–1.060	0.196
**Marital status**	0.970	0.900–1.046	0.432		0.962	0.892–1.038	0.318
**Location**	1.184	1.149–1.219	<0.001		0.971	0.941–1.002	0.064
**Grade**	1.322	1.281–1.365	<0.001		1.301	1.262–1.342	<0.001
**Pathological type**	0.999	0.998–0.999	<0.001		1.000	1.000–1.001	0.702
**Stage (AJCC 8th)**	1.703	1.664–1.743	<0.001		1.239	1.179–1.303	<0.001
**Size**	1.005	1.005–1.006	<0.001		1.004	1.003–1.004	<0.001
**Surgery**	0.638	0.625–0.651	<0.001		0.347	0.327–0.368	<0.001
**Lymph node**	0.894	0.858–0.933	<0.001		1.246	1.190–1.305	<0.001
**Metastasis**	3.032	2.889–3.182	<0.001		1.088	0.978–1.210	0.121
**Radiation**	0.592	0.563–0.621	<0.001		0.865	0.820–0.912	<0.001
**Chemotherapy**	0.538	0.516–0.562	<0.001		0.494	0.471–0.518	<0.001

PSM, propensity score matching; CSS, cancer-specific survival.

### Stratified Analysis of Surgical Treatment

**Figure 2:**
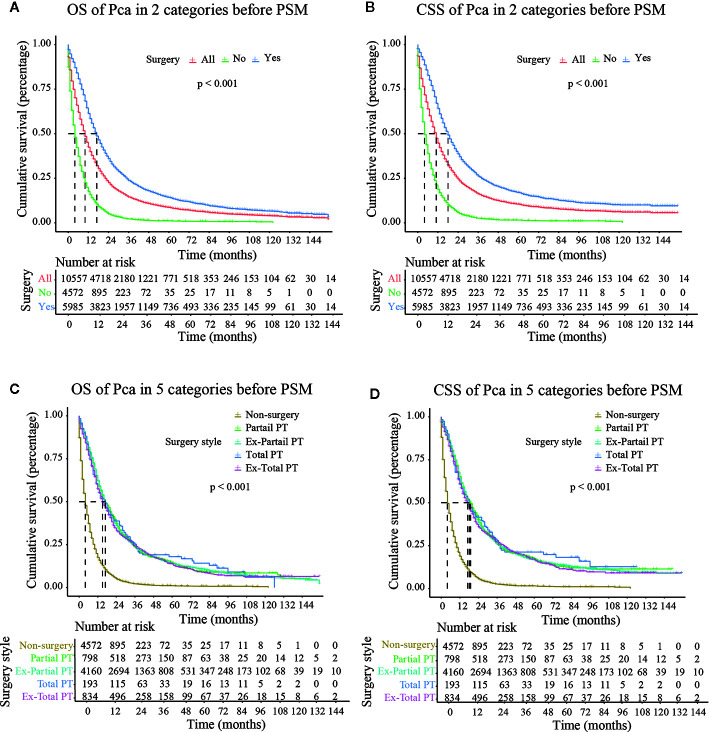
OS (Overall Survival) and CSS (Cancer-Specific Survival) analysis of pancreatic cancer patients in Cohort 2 before PSM (Propensity Score Matching). PT, pancreatectomy; Ex- Partial PT, Extension Partial pancreatectomy; Ex-Total PT, Extension Total pancreatectomy.

**Figure 3:**
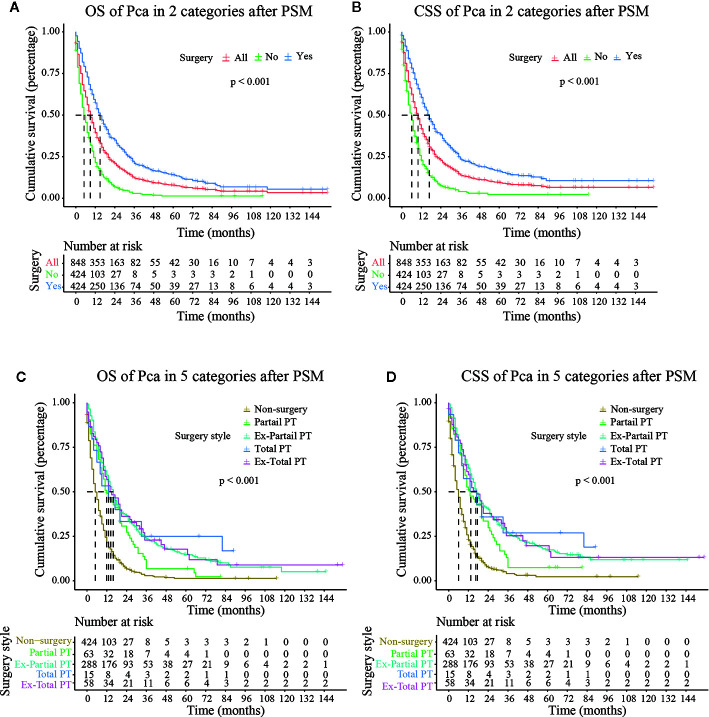
OS (Overall Survival) and CSS (Cancer-Specific Survival) analysis of pancreatic cancer patients in Cohort 2 after PSM (Propensity Score Matching). PT, pancreatectomy; Ex-Partial PT, Extension Partial pancreatectomy; Ex-Total PT, Extension Total pancreatectomy.

The median follow-up time in the non-surgery group and the surgery group was 4.000 months (3.790–4.210) and 17.000 months (16.440–17.560), respectively for OS and 4.000 months (3.770–4.230) and 18.000 months (17.380–18.630), respectively for CSS. Kaplan–Meier survival curves based on OS/CSS before or after PSM are presented in **Figures 2** and **3**. In Cohort 1, the total OS and CSS of the non-surgery group were 7.840 months and 8.490 months, respectively; these values were 31.150 months and 35.950 months, respectively, in the surgery group. The results showed that the total OS and CSS in the surgery group were significantly higher than those in the non-surgery group (*P* < 0.001), indicating that the elderly patients with pancreatic cancer can gain significant survival advantages from surgery. In addition, compared with other surgical methods, elderly patients who underwent Total PT had longer OS and CSS (*P* < 0.001).

As shown in **Figure 3**, after PSM, the median CSS of Partial PT, Ex- Partial PT, Total PT, and Ex-Total PT were 13.000 months (7.988–18.012), 17.000 months (14.738–19.262), 18.000 months (2.539–31.461), and 17.000 months (10.664–23.336), respectively. The results demonstrated that elderly patients who underwent Total PT had the longest median CSS (18.000 months) and greatest survival benefits, while the Ex-Total PT demonstrated a lower median CSS (17.000 months) and median OS (14.000 months). Patients who underwent Partial PT showed the lowest median CSS (13.000 months) and median OS (12.000 months), although these figures were still higher than the median CSS (6.000 months) and median OS (5.000 months) in the non-surgery group.

### Hazard Ratio (HR) in CSS Cox Analysis

**Figure 4:**
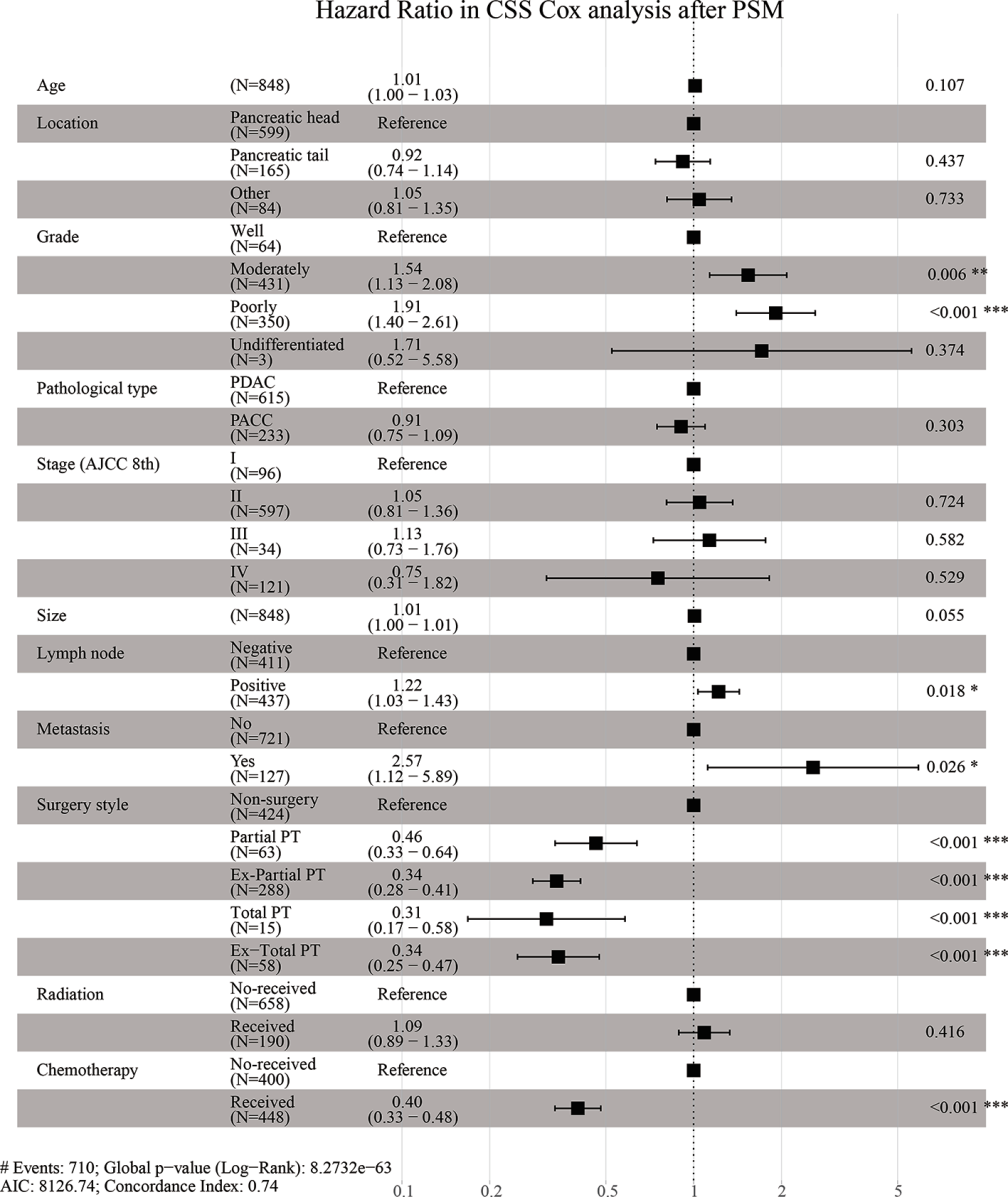
Hazard Ratio in CSS (Cancer-Specific Survival) Cox analysis after PSM (Propensity Score Matching).

**Supplementary Figure 2** and **Figure 4** are forest plots of HRs for CSS Cox analyses in Cohort 1 before and after PSM. As shown in the figures, grade, stage, lymph node metastasis, and distant metastasis were risk factors for CSS in elderly patients with pancreatic cancer, while surgery, radiotherapy, and chemotherapy are protective factors. Among them, the forest plot of Cohort 1 before PSM (**Supplementary Figure 2**) suggested that the HR of Ex-Total PT was lower than those of other surgical methods (HR = 0.32, *P* < 0.001), although the HR value between groups was similar and the difference was small. The forest plot after PSM (**Figure 4**) showed that the HR value gaps between groups with different surgical methods were significantly enlarged, and the elderly patients who underwent Total PT had the lowest risk ratio (HR = 0.31, *P* < 0.001), indicating that these patients had the best survival advantages. These above results suggested that compared with other surgical methods, Total PT can offer elderly patients with pancreatic cancer the best survival advantages. However, Ex-Total PT, a more radical method, was not the best treatment option for the survival and prognosis of elderly patients.

## Discussion

Pancreatic cancer is commonly known as “the king of cancers” and is the deadliest cancer in the Chinese cancer registry, with a survival rate of only 7.2% (19). Due to its features (rapid progression and late detection), pancreatic cancer often occurs in the elderly (20, 21). With the increasing trend of population aging, the numbers of elderly patients with pancreatic cancer will continue to rise. Surgery is considered the main treatment modality for pancreatic cancer (22). However, many elderly patients refuse surgery due to factors such as age and physical condition. As a result, only few patients undergo surgery (23). Nowadays, surgery plays an indispensable role in the treatment of pancreatic cancer, but it is not clear whether elderly patients can gain survival advantages from more active and radical surgical treatment. Therefore, it is challenging to find better treatment method for elderly patients with pancreatic cancer.

In this study, patients who underwent surgical procedures, including Partial PT, Ex-Partial PT, Total PT, and Ex-Total PT, had a higher survival rate than the non-surgery group. This is consistent with the results of previous studies (24). Wegner et al. (25) found that postoperative mortality in all age groups was increasing and that it increased with the scope of surgery. A multi-center study by Sho et al. (26) demonstrated that due to the reduced completion rate of adjuvant chemotherapy, the prognosis of patients above the age of 80 years was not as good as that of younger patients. However, Renz et al. (27) collected and analyzed the data of 300 patients who underwent partial pancreaticoduodenectomy (PD) or pylorus-preserving pancreaticoduodenectomy (PPPD) from 2002 to 2012. They found that patients above the age of 75 years had similar prognoses with younger patients, and there were no differences in perioperative mortality, surgical complications, and OS. This result implied that age should not be a limiting factor for surgery in elderly patients with pancreatic cancer, which was also consistent with the results of a study by Higuera et al. (28).

The Kaplan–Meier survival analysis of OS and CSS after PSM in this study showed that the average and median CSS of Ex-Partial PT were 35.864 and 17.000 months, respectively, and those for total PT were 32.667 months and 18.000 months, respectively. It could be seen that the average CSS for Ex-Partial PT was higher, but the median CSS was lower than that for Total PT. Considering that survival data analysis often has a truncation phenomenon that affects the results (29), and the study by Lousdal et al. (30) indicated that it was reasonable to determine prognosis based on the median survival time for cancers. Therefore, in terms of the results of this study, the median CSS for determining prognosis is valuable and reasonable. In addition, Stoop et al. (31) reported that Total PT could be a better treatment option for elderly patients with pancreatic cancer. However, Johnston et al. (32) studied the clinical data of 2,582 patients and found that the mortality rate of Total PT was higher than those in previous reports, and the survival time after surgery was limited. This result could be related to the fact that the average CSS of Total PT in this study was not the highest. Based on the analysis of the National Cancer Database of the United States, Passeri et al. (33) found that there was no significant difference in the survival of patients who underwent Ex-Total PT and that of patients who underwent Total PT. However, Ex-Total PT showed a lower mean CSS (17.000 months) and median OS (14.000 months) than Total PT in this study. This was in line with the findings of the study by Hartwig et al. (34), who reported that Ex-Total PT was associated with increased perioperative incidence and mortality. This suggested that we could not blindly pursue the expansion of the scope of surgery. In recent years, there have been more and more reports on methods to optimize pancreatic cancer surgery, such as spleen-preserving pancreatectomy using Warshaw technology and minimally invasive (laparoscopic or robot-assisted) surgery (35). Clinicians need to combine advanced technology to comprehensively evaluate the possibility of surgery in terms of comorbidities, cognitive status, and preoperative functional status. On the one hand, elderly patients with pancreatic cancer should not be exempted from surgery because of their age. In addition, the scope of surgery should not be blindly expanded.

Nowadays, choosing the best treatment modality for elderly patients with pancreatic cancer is still challenging, and more relevant studies are required to discuss this issue. The results of this study indicated that surgery was beneficial to the survival of elderly patients with pancreatic cancer, and that Total PT might offer them the best survival advantages. However, surgery alone is not enough, as more than 90% of patients relapse and die of the disease after surgery without additional treatment (36). Radiotherapy, chemotherapy, and surgery were shown to be independent factors affecting survival and prognosis in this study. The randomized controlled trial by Macedo et al. (37) showed that OS and disease-free survival of patients with pancreatic cancer were improved by the completion of adjuvant chemotherapy based on gemcitabine. In addition, the combined model of surgery within 4–8 weeks after radiotherapy has been recommended in the 2019 National Comprehensive Cancer Network Guidelines (38). The addition of radiotherapy to neoadjuvant chemotherapy had also been confirmed to be associated with improvements in anti-tumor efficacy and surgical resection (R0 rate) (39). Due to the high complexity and mortality associated with pancreatic cancer, it is extremely controversial to perform surgery on elderly patients, which also reminds us of the importance of a comprehensive treatment in elderly patients with pancreatic cancer. In short, the comprehensive therapy with surgery combined with chemotherapy or radiotherapy might be beneficial in improving the long-term prognosis of elderly patients with pancreatic cancer. However, the optimal combination of surgery, chemotherapy, and radiotherapy needs to be studied further.

In order to increase credibility, this study rigorously selected data sources, set exclusion criteria, and adopted a public and high-quality SEER database. Patients whose pancreatic cancer was neither the only nor the first tumor and those without complete clinical data were excluded. To adjust for confounding variables and reduce treatment selection bias, this study also conducted PSM analysis. PSM summarized the characteristics of all patients into a single covariate and reduced the possibility of overfitting (40). Therefore, in the absence of other forms of bias, systemic differences in outcomes between treatment groups can be attributed to the treatment method (41). After PSM, the differences in outcomes were more prominent, and the results could be better presented.

Of course, this study also has certain limitations. PSM can only balance observable confounding factors, while residual deviation is still unavoidable. Also, individuals that could be matched were excluded, resulting in a decrease in sample size and accuracy. Besides, The SEER database has a considerable number of missing observations, and the dependence on medical diagnosis and program coding could cause huge variability and taint the accuracy of information (42). Scholten et al. (43) used the EORTC QLQ-C30 questionnaire to assess the overall quality of life of 1,536 pancreatic cancer patients and found that the overall quality of life decreased after Total PT. However, due to the retrospective design of this study, the quality of life of patients with pancreatic cancer could not be assessed. Furthermore, the SEER database could not reflect the chronological relationship between surgery, radiotherapy, and chemotherapy; so it was impossible to deeply analyze the impact of surgery combined with other treatment modalities on the prognosis of these patients. In addition, the prognosis of elderly patients with pancreatic cancer is affected by multiple factors. Previous studies have confirmed that the prognosis of pancreatic cancer was closely associated with lifestyle (including smoking, drinking), family income, metabolic diseases (diabetes, obesity), among others (5). The World Cancer Research Center’s research on carcinogenic factors showed that long-term smoking could increase the risk of pancreatic cancer. The proportion of smokers was much higher than that of non-smokers, and there was a dose–response relationship. Moreover, pancreatic cancer is also associated with alcohol consumption. A study demonstrated that men with a weekly alcohol intake of more than 420 g had a 70% higher risk of pancreatic cancer (44). The economic burden of pancreatic cancer is huge for every family. Family income differences make different individuals choose different lifestyles and treatment methods, and the development process of cancer also changes accordingly (45). In this study, we further analyzed the relationship between medical insurance and the prognosis of elderly patients with pancreatic cancer. After excluding patients with incomplete information, a total of 8,439 patients were retained (medical insurance: 8,381, no medical insurance: 58). The number of patients who underwent surgery was 4,805 and 33, respectively. The mean CSS of the two groups was 16.132 months and 16.363 months. We found that there was no significant difference in CSS between medical insurance and no medical insurance (*P* < 0.05), which was consistent with the conclusion of Sridhar et al. (46). This could be due to the small number of people without medical insurance in our study. In addition, Cascetta et al. (47) proved that pancreatic fat fraction was negatively correlated with the survival of PDAC patients. There is a mutual relationship between pancreatic cancer and diabetes. Diabetes is not only one of the causes of pancreatic cancer but also one of the complications of pancreatic cancer. Ma J et al. (48) found that patients with diabetes had significantly lower survival rates, larger tumors, and higher risks of death. The metastatic location of pancreatic cancer is also closely associated with prognosis. Oweira et al. (49) found that patients with isolated distant lymph nodes or lung metastases had higher OS and CSS than liver metastases. However, limited by the paucity of information in the SEER database, our study failed to assess whether the above factors affect the survival of elderly patients with pancreatic cancer, which might reduce the accuracy and comprehensiveness of the results.

## Conclusion

In a nutshell, surgery is an independent prognostic factor, and it is beneficial to the survival of elderly patients with pancreatic cancer. Compared with other surgical methods, Total PT might offer elderly patients the best survival advantages. However, more radical Ex-Total PT does not seem to be the best treatment option for elderly patients with pancreatic cancer.

## Data Availability Statement

Publicly available datasets were analyzed in this study. This data can be found here: Surveillance, Epidemiology, and End Results (SEER) database (https://seer.cancer.gov/).

## Ethics Statement

Ethical review and approval was not required for the study on human participants in accordance with the local legislation and institutional requirements. Written informed consent for participation was not required for this study in accordance with the national legislation and the institutional requirements. The ethics committee waived the requirement of written informed consent for participation.

## Author Contributions

(I) Conception and design: YH. (II) Administrative support: YH. (III) Collection and assembly of data: DX, BQ, YL, TH, SL, XC. (V) Data analysis and interpretation: DX, BQ, JY, XP. All authors contributed to the article and approved the submitted version.

## Funding

This work was supported by a grant from Project of Science and Technology Department of Sichuan Province (2020JDTD0036), Beijing Medical and Health Foundation (YWJKJJHKYJJ-F3229D) and the Talent development project of The Affiliated Hospital of Southwest Medical University.

## Conflict of Interest

The authors declare that the research was conducted in the absence of any commercial or financial relationships that could be construed as a potential conflict of interest.
