# Supplementary material for: Can Elderly Patients With Pancreatic Cancer Gain Survival Advantages Through More Radical Surgeries? A SEER-Based Analysis
Source: Front Oncol. 2020 Oct 29;10:598048. doi: 10.3389/fonc.2020.598048 (PMC7660699; doi:10.3389/fonc.2020.598048)
Supplement: Supplementary file 1 [file Table_1.docx]

Supplementary Material

# Supplementary table 1 Baseline characteristics after PSM.

| **Term** | **No. of Patients (%)** | | ***P*-value** |
| --- | --- | --- | --- |
|  | Non-surgery（n=424） | Surgery（n=424） |  |
| **Age (years)** |  |  | 0.572 |
| Mean (SD) | 73.63 (6.01) | 73.87 (6.00) |  |
| **Gender** |  |  | 1.000 |
| Female | 229 (54.0) | 228 (53.8) |  |
| **Race** |  |  | 0.315 |
| White | 348 (82.1) | 361 (85.1) |  |
| Black | 41 (9.7) | 29 (6.8) |  |
| Other | 35 (8.3) | 34 (8.0) |  |
| **Marital status** |  |  | 0.232 |
| Married | 380 (89.6) | 391 (92.2) |  |
| **Location** |  |  | 0.378 |
| Pancreatic head | 296 (69.8) | 303 (71.5) |  |
| Pancreatic tail | 80 (18.9) | 85 (20.0) |  |
| Other | 48 (11.3) | 36 (8.5) |  |
| **Grade** |  |  | 0.220 |
| Well | 35 (8.3) | 29 (6.8) |  |
| Moderately | 207 (48.8) | 224 (52.8) |  |
| Poorly | 179 (42.2) | 171 (40.3) |  |
| Undifferentiated | 3 (0.7) | 0 (0.0) |  |
| **Pathological type** |  |  | 0.001 |
| PDAC | 388 (91.5) | 227 (53.5) |  |
| PACC | 36 (8.5) | 197 (46.5) |  |
| **Stage (AJCC 8th)** |  |  | 0.449 |
| I | 54 (12.7) | 42 (9.9) |  |
| II | 289 (68.2) | 308 (72.6) |  |
| III | 19 (4.5) | 15 (3.5) |  |
| IV | 62 (14.6) | 59 (13.9) |  |
| **Size** |  |  | 0.664 |
| Mean (SD) | 35.96 (11.71) | 35.61 (11.65) |  |
| **Lymph node** |  |  | 0.891 |
| Positive | 217 (51.2) | 220 (51.9) |  |
| **Metastasis** |  |  | 0.700 |
| Positive | 66 (15.6) | 61 (14.4) |  |
| **Radiation** |  |  | 0.680 |
| Received | 92 (21.7) | 98 (23.1) |  |
| **Chemotherapy** |  |  | 0.945 |
| Received | 225 (53.1) | 223 (52.6) |  |

PSM, Propensity Score Matching; PDAC, pancreatic ductal adenocarcinoma; PACC, pancreatic acinar cell carcinoma; SD, Standard deviation.

# Supplementary figure 1


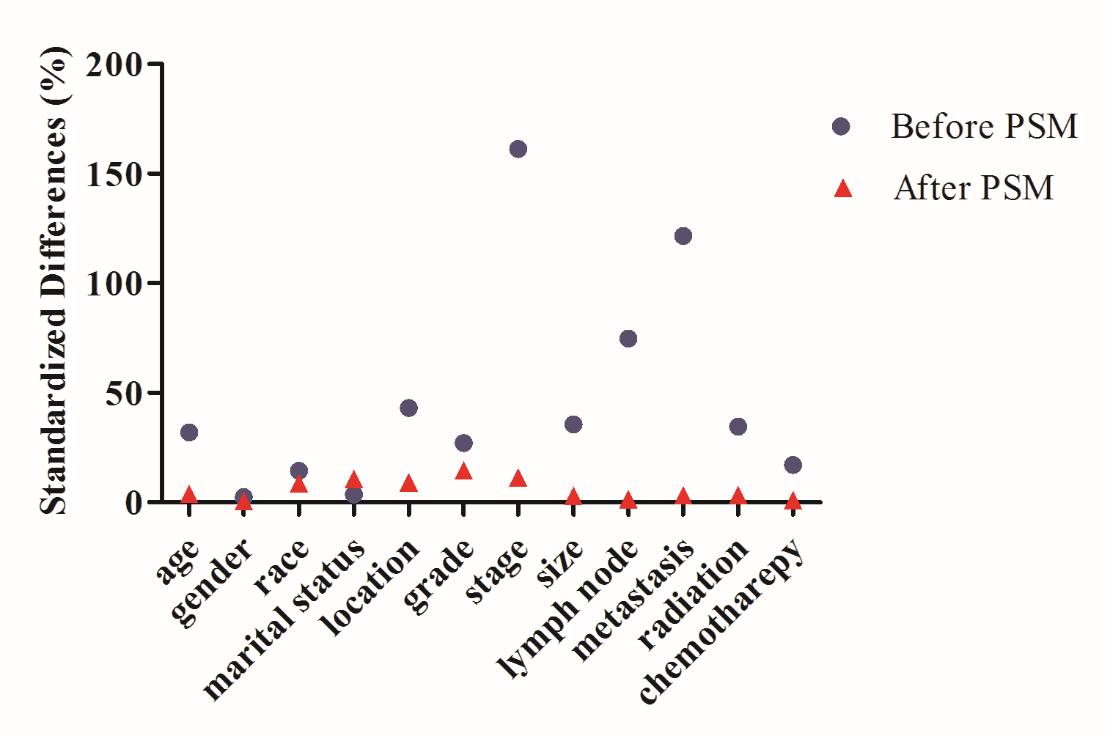


**Supplementary figure 1.** Standardized differences before and after PSM. PSM, Propensity Score Matching.

# Supplementary figure 2


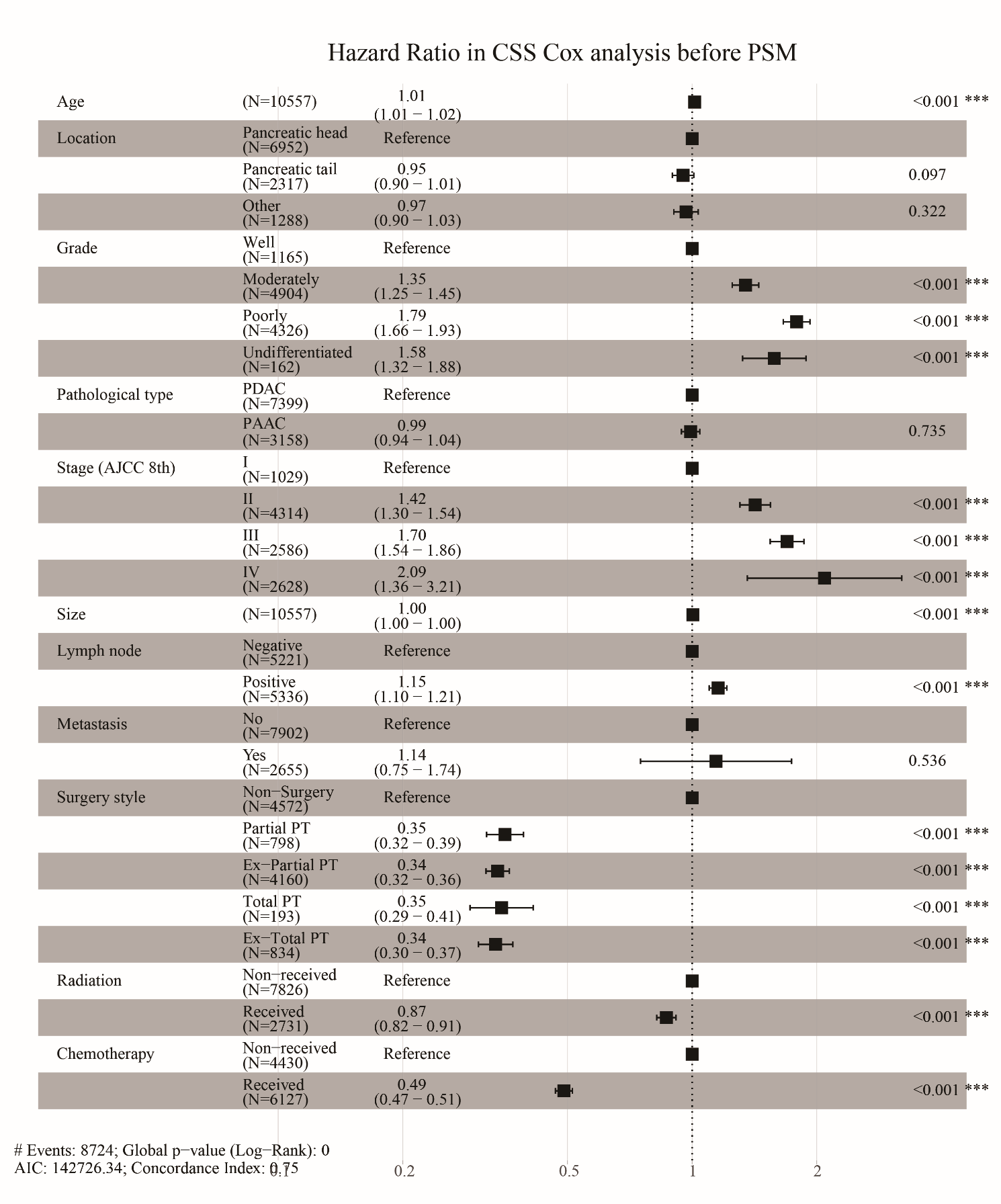


**Supplementary figure 2.** Hazard Ratio in CSS (Cancer-Specific Survival) Cox analysis before PSM (Propensity Score Matching).
